# Methicillin-resistant *Staphylococcus aureus*-associated empyema necessitans in a child: A case report and a literature review

**DOI:** 10.5339/qmj.2024.35

**Published:** 2024-07-01

**Authors:** Ghada Habachi, Sondes Sahli, Sabrine Ben Ammar, Bochra Aziza, Riadh Jouini

**Affiliations:** 1 Department of Pediatric Surgery “A”, Children’s Hospital of Tunis, Tunis, Tunisia *Email: habachighada92@gmail.com

**Keywords:** Children, empyema necessitans, MRSA

## Abstract

**Introduction:**

Empyema is a known complication of severe pleuropneumonia. In rare cases, if poorly treated, it could result in dissemination and fistulization and transformation into empyema necessitans. The manifestation may appear as a superficial abscess. However, as management highly differs, the recognition of potentially severe phenomenon is required.

**Case Presentation:**

We describe a case of empyema necessitans on a 4-year-old girl secondary to methicillin-resistant *Staphylococcus aureus*. It represents the sixth pediatric case reported in the literature. It was managed by open drainage and prolonged antibiotherapy. The outcome was favorable as guidelines were extracted from similar reported cases.

**Conclusion:**

Empyema necessitans remains a rare complication with significant morbidity. Prompt diagnosis and adapted management have relied on limited literature. As such, further reports are necessary to establish proper guidelines.

## Introduction

Empyema is a well-known complication of pleuroneumonia. It complicates up to 5% of community-acquired pneumonia in children.^1^ It is secondary to the transformation of exsudative parapneumic effusion into frank pus with the disposition of fibrin and cellular material.^2^ Empyema necessitans or empyema necessitatis (EN) represents a rare long-term complication of poorly or uncontrolled thoracic empyema in which purulence dissects through the thoracic wall and diffuses through the soft tissue.^3^ Under pressure, it may dissipate into the skin, the mediastinum, the bronchus, the esophagus, or the diaphragm.

Since first described in 1640 by Gullan de Billon as a syphilitic aneurysm ruptured through the chest wall, numerous cases have been identified mainly in adults, with a high mortality rate approaching 20% of cases.^4,5^ However, in children, it remains rare and more benign due to its superior healing potential. Therefore, the treatment differs significantly with a constant debate over the operative versus the non-operative approach.^6^

**Figure 1.**
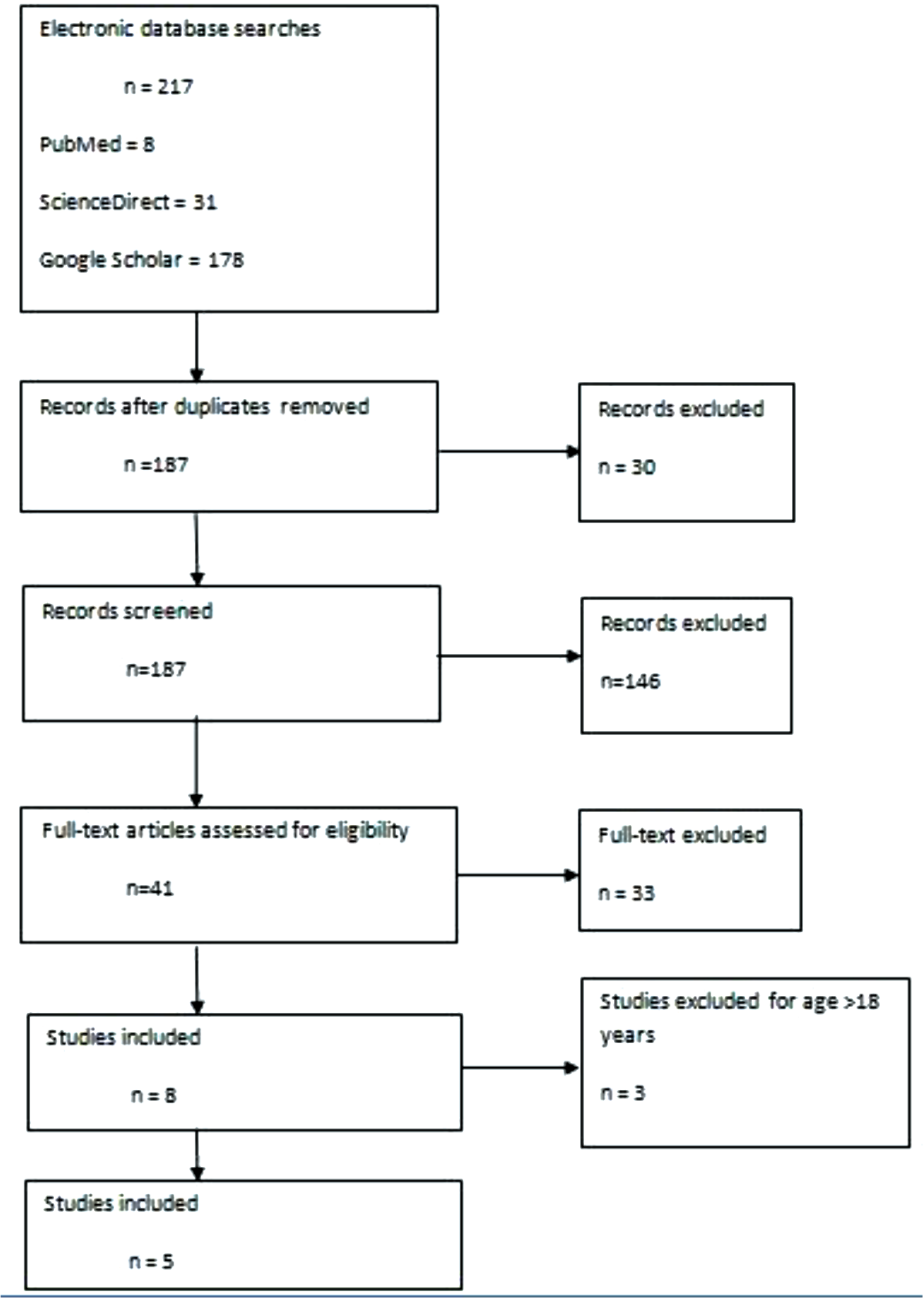
PRISMA flow diagram of the literature review process for methicillin-resistant *Staphylococcus aureus* (MRSA)-associated empyema necessitans in children.

Pleural effusion complicated with EN has been linked primarily to *Mycobacterium tuberculosis* and *Actinomyces israelii*.^2^ Although other microbial pathogens have been reported including *Streptococcus pneumoniae*, *Escherichia coli*, *Pseudomonas cepacia*, *Klebsiella pneumoniae*, and anaerobes.^7^ Recently, methicillin-resistant *Staphylococcus aureus* (MRSA) has emerged as an increasing cause of severe infections.^8^ However, in the case of EN and despite its widespread distribution, it has been rarely reported in both adults and children.^7^ A systemic literature review was conducted using PubMed, ScienceDirect, and Google Scholar databases. The keywords used were “empyema necessitans,” empyema necessitates,” “MRSA,” and “children” with no logic operators returning 217 papers. After removing non-relevant papers and reviews, eight cases were identified, five of which were children (Figure 1).^3,9–12^ Due to a scarcity of literature, the majority of pediatric practice derives from adult studies, and exact guidelines have yet to be determined.

Herein, we describe a case of empyema necessitans on a 4-year-old girl secondary to MRSA. As the sixth pediatric case, ours highlights a rare causative microbiologic agent. It also represents the first reported case in Tunisia. As data are scarce, we conducted a literature review to develop a management strategy for children. The patient and his or her parents provided informed consent for publication.

## Case Presentation

A 4-year-old girl, with no significant past medical history, was referred to our governmental surgical center by her primary care pediatrician with complaints of thoracic wall mass for the previous 2 months. She had no history of trauma and reported being previously hospitalized 12 months prior for pneumonia which required a 1-week hospital stay and was treated with intravenous antibiotherapy but no oxygenotherapy.

**Figure 2.**
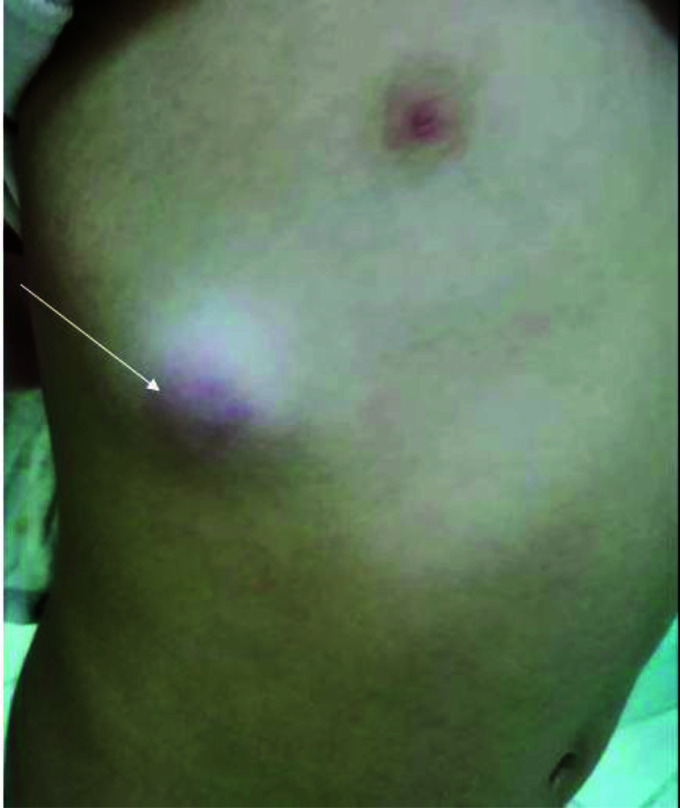
A picture of the child’s chest showing empyema necessitans (arrowhead).

Currently, she is asymptomatic with no fever and no respiratory symptoms. Her physical examination revealed normal bilateral breath sounds but revealed a 4×5 cm firm and tender thoracic swelling with overlying erythema located between the right midclavicular and axillary lines, approximately in the seventh intercostal space (Figure 2). The remainder of her physical examination was unremarkable.

Laboratory data showed a normal white blood cell count with 35% neutrophils, 5% eosinophils, 48% basophils, 0.7% lymphocytes, and 11.3% monocytes. The hemoglobin level and platelet counts were both normal, and the C-reactive protein level was negative. Chest X-ray revealed an obliteration of the right costophrenic angle with no lung or bone infiltration associated with a soft tissue swelling of the right chest wall.

**Figure 3.**
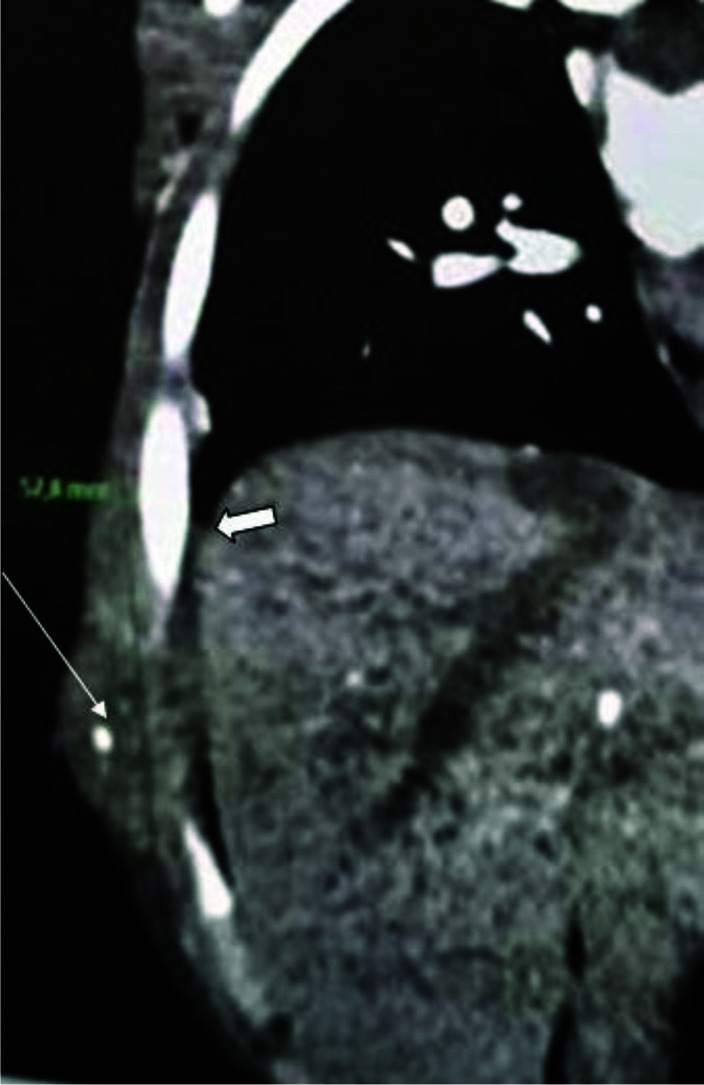
Coronal thoraco-abdominal computed tomography with a mediastinal window showing a 5×6 cm empyema eroding into the right anterior chest wall (arrowhead) associated with pleural densification (large arrow) confirming the diagnosis of empyema necessitans.

Given the clinical and radiological findings, a panel of diagnoses was considered, including rhabdomyosarcoma, Ewing sarcoma, and tuberculous empyema necessitans. Contrast computed tomography (CT) revealed a 5×6 cm empyema eroding into the chest wall with visualization of a pleural fistula and with no associated pleural effusion (Figure 3). The diagnosis of empyema necessitans was thus confirmed.

The patient was isolated considering the possibility of tuberculosis and three sputum samples induced by nebulized sterile saline, and blood samples were sought for mycobacterial and Gram cultures that returned negative. The option of chest tube drainage was ruled out given the lack of pleural effusion. An open aspiration and drainage was made producing frank pus which grew MRSA. The isolate was sensitive to clindamycin, gentamycin, rifampin, trimethoprim-sulfamethoxazole (TMP-SMX), vancomycin, and teicoplanin. She was treated with a 5-day course of intravenous gentamycin and a 10-day course of intravenous teicoplanin given a vancomycin allergy. She was discharged after 14 days of hospital stay and continued oral TMP-SMX for another 4 weeks. Consequently, the total duration of antibiotherapy was 40 days.

Immunological studies showed no deficiencies as neutrophil and lymphocyte numbers were normal, as were the nitroblue tetrazolium test and quantitative serum immunoglobulin levels. Eight weeks later, follow-up ultrasonography revealed complete resolution of the empyema and obliteration of the pleural fistula. Twelve months later, the patient is recovering well with no evidence of recurrence.

## Discussion

Empyema necessitans is a rare phenomenon secondary to the insidious extension of a persistent infected parapneumonic fluid through the adjacent subcutaneous tissue and into the skin.^13^ Usually, the perforation is located at the anterior chest wall due to the frequent adhesions along the apical, basilar, and posterior lung.^3,5^ This was demonstrated in our case as the perforation occurred on the seventh intercostal space along the anterior chest wall. Nevertheless, in rare cases, the perforation could extend toward the opposite pathway into the controlateral chest wall or even into the abdominal and cervical spaces.^14,15^ The discovery of a chest wall mass poses a diagnostic quandary as differential diagnosis includes inflammatory, infectious, and neoplastic conditions.^10^ Thoracic X-ray may be normal or reveal a soft tissue density in the chest wall and may help in the detection of associated pneumatic pathology. Ultrasound represents a quick tool that orients the physician. Though limited by the ribcage, it may show a heterogeneous parietal mass and seldom may it aid in visualizing the communication between the pulmonary infection and the anterior chest wall.^13,16^ Subsequently, CT represents the recommended imaging modality for diagnosing this condition, including in children.^7^ Even though it is associated with a high risk of radiation, recent innovations and appropriate settings allow for lower doses for children nowadays.

Multiple etiologies have been linked to this complication including trauma,^17^ recent thoracotomy,^13^ necrotizing pneumonia,^18^ and immune deficiency,^19^ with the untreated pleural effusion being the most common etiology in 70% of cases.^4^ Moreover, breastfeeding has been documented as a risk factor in a neonate due to the presence of severe mastitis and breast abscess in the mother.^11^ In our case, the child was fully vaccinated with no chronic illness and no underlying deficiency. We believe that her condition is secondary to a badly treated pneumonia 12 months prior.

In 75% of cases, fibrinous debris and bacteria such as *M. tuberculosis* and *A. israelii* accumulate in the effusion.^20^ This case describes an uncommon microbiologic cause of EN. As reported in our case, MRSA had previously only been identified in five pediatric cases (Table 1).^3,9–12^ The ages ranged from 1 month to 5 years. Our case is the second case in the literature to be over 2 years old. It is also the first pediatric case to be reported in Tunisia.

Because our patient had been hospitalized within the previous year, we were unable to distinguish this case as a community or health-care-associated MRSA. Furthermore, our facility lacks the necessary resources to distinguish the strains phenotypically and genotypically.^10^ Despite the rapid rise in infections caused by this bacterium due to the overuse of antibiotics, only six cases of MRSA can be identified. This could also be a result of the empiric use of antibiotics and the rise of falsely negative bacteriological cultures.^8,21^

Given the scarcity of case reports, no guidelines for children have been established. The majority of practice is extrapolated from adult data. The management relies on early diagnosis, appropriate drainage, and vigorous antibiotherapy. To facilitate lung expansion, the drainage should be extensive, with thoracoscopic decortication or the insertion of a chest tube as needed.^2,22^ In our case, we opted for open drainage because there was no associated pleural effusion and no adhesions on the CT.

The antibiotherapy should be based on the antibiogram and should be started right after acquiring a biological specimen. However, the exact duration has yet to be determined. Based on this review, we opted for a 10-day intravenous treatment followed by 4 weeks of TMP-SMX (Table 1). Nevertheless, in severe cases, prolonged intravenous therapy should be prescribed.^2,4^ Vancomycin represents the primary therapy in the literature; however, in our case, an allergic reaction compelled us to switch to teicoplanin, which has similar efficacy.^23,24^

Outcomes are usually favorable when the diagnosis is made early and management is adapted to the causative agent. Thus, a high index of suspicion is required.

## Conclusion

MRSA-associated empyema necessitans is a rarely reported disease in children. Only five previous case reports have been identified in the published literature. As such, protocols are lacking and data are scarce. They are based on previously reported cases. The management should combine adequate drainage and antibiotherapy. Outcomes are favorable in the case of early diagnosis and management. A high index of suspicion is necessary to prevent this rare evolution, and additional reports are required to promote diagnostic and therapeutic guidelines.

## Patient Consent Statement

Informed consent was received from the patient and the parents.

## Ethics Approval and Consent to Participate

Not applicable.

## Consent for Publication

The patient’s approval for publication has been obtained.

## Conflict of Interest Statement

The authors declare that they have no competing interests.

## Data Availability Statement

Data sharing is not applicable to this article as no data-sets were generated or analyzed during this study.

## Authors’ Contributions

GH participated in the investigation and writing of the original draft. SS participated in the investigation, editing, and rewriting of the draft. SBA participated in the investigation. BA participated in reviewing and editing. RJ participated in the project administration, supervision, and visualization. All authors read and approved the final manuscript.

## Acknowledgments

Not applicable.

## Figures and Tables

**Table 1.** Reported pediatric cases of methicillin-resistant Staphylococcus aureus associated empyema necessitans.

**Study/year**	**Age**	**Surgical procedure**	**Intravenous antibiotherapy**	**Oral antibiotherapy**
Stallworth et al.^9^	8 months	Chest tube	Vancomycin → 10 days	Trimethoprim-sulfamethoxazole (TMP-SMX) → 21 days
Moore^3^	3 months	Thoracotomy + decortications + subscapular drainage	Vancomycin → 14 days	Linezolid → 7 days
Contreras et al.^10^	19 months	Thoracoscopic decortication	Vancomycin → 14 days + Gentamycin (associated osteomyelitis)	Vancomycin + clindamycin → 24 days
Rosebush et al.^11^	1 month	Pigtail catheter drainage	Clindamycin → 4 weeks	Clindamycin → 4 weeks
Pugh^12^	5 years	Chest tube drainage + pleural fibrinolytic agents in video-assisted thoracoscopy	Clindamycin → 21 days	None
Our case (2023)	4 years	Open drainage	Teicoplanin → 10 days + gentamycin	TMP-SMX → 30 days
